# A CRISPR CASe for high-throughput silencing

**DOI:** 10.3389/fgene.2013.00193

**Published:** 2013-10-07

**Authors:** Jacob Heintze, Christin Luft, Robin Ketteler

**Affiliations:** Medical Research Council Laboratory for Molecular Cell Biology, University College LondonLondon, UK

**Keywords:** CRISPR, Cas9, RNAi, gene silencing, gene editing, knockdown, screen, high-throughput

## Abstract

Manipulation of gene expression on a genome-wide level is one of the most important systematic tools in the post-genome era. Such manipulations have largely been enabled by expression cloning approaches using sequence-verified cDNA libraries, large-scale RNA interference libraries (shRNA or siRNA) and zinc finger nuclease technologies. More recently, the CRISPR (clustered regularly interspaced short palindromic repeats) and CRISPR-associated (Cas)9-mediated gene editing technology has been described that holds great promise for future use of this technology in genomic manipulation. It was suggested that the CRISPR system has the potential to be used in high-throughput, large-scale loss of function screening. Here we discuss some of the challenges in engineering of CRISPR/Cas genomic libraries and some of the aspects that need to be addressed in order to use this technology on a high-throughput scale.

## BACKGROUND

Advances in lab automation and completion of the human genome project have resulted in the design of large-scale whole-genome libraries encompassing thousands of samples in a single run. These developments build on technological advances made by the pharmaceutical industry that required a need for screening large collections of chemical compounds in a very cost-effective manner and were subsequently adopted by academic institutions for chemical compound screening as well as expression cloning applications. Such large-scale screening approaches have enabled the unbiased identification of cell-based phenotypes and led to several ground-breaking discoveries, in particular for the identification of cell surface and virus receptors (Seed, 1995).

Expression cloning relies on the generation of an expressed cDNA library that can be transfected into target cells for assessment of changes in a cellular reporter activity or phenotype. Over the years, a complementary approach was developed based on the use of “antisense” constructs without a proper understanding of the underlying mechanisms. Eventually, after the discovery of RNA interference in the late 1990s and the development of mammalian siRNA libraries in the early 2000s, such knockdown technologies became mainstream for interrogating gene defects on a genome-wide scale. To date, siRNA genome-wide libraries are still the main application in genomic high-throughput screening, partially because of the lack of alternative methods. In recent years, many key problems of RNAi technology have become apparent, such as off-target effects, variable levels of knockdown efficiency and low-level confidence in hits of screening campaigns (Buehler et al., 2012; Marine et al., 2012). Hence, the siRNA screening community is openly discussing the need for alternative systems to overcome these limitations.

An alternative way of modifying the mammalian genome on a large-scale may lie in the use of genome editing technologies such as the use of targeted endonucleases. While zinc finger nucleases (ZFNs) and transcription activator-like effector nuclease (TALEN)s are highly useful for gene editing, the cost and time to engineer the system makes it incompatible for the generation of large-scale whole-genome libraries. In particular, ZFN or TALENs require engineering of a protein component for each gene locus and this may not be amenable or cost-effective on a large-scale. With the CRISPR (clustered regularly interspaced short palindromic repeats)/Cas9 system, a cost-effective method that combines targeted genome editing with a simple, less time-consuming assay set-up is emerging and may be compatible with high-throughput screening approaches. An overview of the main techniques for genome editing and gene silencing is given in **Table 1** and a detailed assessment of the advantages and disadvantages of genome editing methods has been given elsewhere (Ramalingam et al., 2013).

**Table 1:** Overview of genome editing and gene silencing technologies.

Name	Components	Mechanism of action	Specificity/off-target effects	Possibility to rapidly generate large-scale libraries
**Genome editing**				
Zinc finger nucleases (ZFNs)	Fok1 restriction nuclease fused to multiple zinc finger peptides; each targeting 3 bp of genomic sequence	Induces double-strand breaks in target DNA	Can have off-target effects	No – requires customization of protein component for each gene
Transcription activator-like effector nucleases (TALENs)	Non-specific DNA-cleaving nuclease fused to a DNA-binding domain specific for a genomic locus	Induces double-strand breaks in target DNA	Highly specific	Feasible, but technically challenging (Reyon etal., 2012)
Homing meganucleases	Endonuclease with a large recognition site for DNA (12–40 base pairs)	Induces double-strand breaks in target DNA	Highly specific	No – limited target sequence specificity available
CRISPR/Cas	20 nt crRNA fused to tracrRNA and Cas9 endonuclease	Induces double-strand breaks in target DNA (wt Cas9) or single-strand DNA nicks (Cas9 nickase)	Some off-target effects that can be minimized by selection of unique crRNA sequences	Yes – requires simple adapter cloning of 20 nt Oligos targeting each gene into a plasmid
**Gene silencing**				
Post-transcriptional gene silencing (e.g., RNA interference)	Double-stranded RNA	DICER-mediated mRNA degradation; (post-transcriptional)	Can have significant off-target effects	Yes (Moffat etal., 2006)
Morpholino oligonucleotides	Synthetic oligonucleotide analogs	Sterical blocking of translation initiation complex; (post-transcriptional)	Can have significant off-target effects	Feasible, but technically challenging
CRISPRi	sgRNA and catalytically inactive Cas9	Transcriptional repression of RNA synthesis	To be determined	Yes

## CRISPR/Cas9

The CRISPR/Cas9 system, originally exploited from *Streptococcus pyogenes*, is a general bacterial host defense mechanism to detect and degrade exogenous sequences from invading bacteriophages (Sorek et al., 2013). Short segments of foreign DNA are processed by CRISPR-associated (Cas) proteins into small elements which are then inserted into the CRISPR locus. RNAs from the CRISPR loci are constitutively transcribed and processed into small RNAs (crRNA) of exogenously derived DNA. These small RNAs then guide other Cas proteins such as Cas9 to mediate sequence-specific degradation of the foreign DNA. As such, the system serves two critical functions of acquired immunity, memory and pathogen elimination, and has some resemblance with acquired immunity in higher organisms. This system relies on the DNA endonuclease Cas9 that is targeted to a specific region of the genome to direct cleavage of the DNA in a sequence-specific manner (Jinek et al., 2012). Binding of Cas9 to the DNA is mediated by a partially complementary *trans*-acting RNA (tracrRNA) and cleavage is directed by a mature CRISPR RNA (crRNA; Deltcheva et al., 2011; Jinek et al., 2012). These three components, the Cas endonuclease, the tracrRNA, and crRNA are the basic constituents of this genome editing system.

By modifying these basic constituents for the use in other organisms, the CRISPR/Cas9 system has been shown to be a useful tool for gene editing and silencing. CRISPR sequences can be engineered which give rise to crRNA directed against specific endogenous genes in various organisms. Significant advances for the use of this technique have been promoted by the generation of a “single-guide RNA” (sgRNA) that combines the function of the tracrRNA and crRNA in a chimeric molecule (Jinek et al., 2012, 2013). By now the CRISPR/Cas9 system has been successfully used to target genomic loci in mammalian cell lines (Cho et al., 2013; Cong et al., 2013; Mali et al., 2013; Wang et al., 2013), zebrafish (Chang et al., 2013; Hwang et al., 2013), fungi (Dicarlo et al., 2013), bacteria (Jiang et al., 2013; Mali et al., 2013), *C. elegans* (Friedland et al., 2013), *Drosophila* (Gratz et al., 2013), plants (Li et al., 2013b; Nekrasov et al., 2013; Shan et al., 2013), rats (Li et al., 2013a, c), and mice (Wang et al., 2013). For instance, gene knockout mice can be rapidly generated when the desired gene locus is targeted via CRISPR/Cas9 (Wang et al., 2013). Quite importantly, multiple loci can be targeted at the same time by incorporation of multiple crRNA sequences (Cho et al., 2013; Wang et al., 2013). In zebrafish, where RNAi-based methods have limited capability, the use of CRISPR has enabled the generation of whole animals deficient in multiple gene loci (Chang et al., 2013; Hwang et al., 2013). The same system has been used to site-specifically insert mloxP sites, making it a novel reverse genetic tool for genome modification (Chang et al., 2013). In zebrafish and *Drosophila* it has been shown that CRISPR/Cas9 genome editing is inheritable with germline transmission reaching nearly 100% (Basset et al., 2013; Hwang et al., 2013; Yu et al., 2013).

Up to date three different versions of the Cas9 component have been described for the use in mammalian cells.

(A) Wild-type humanized (h)Cas9: Wild-type Cas9 will introduce a double-strand break (DSB) at the region it is targeted to, thus resulting in activation of the DSB repair machinery. Consequently, this will lead to insertion or deletion of nucleotides at the site of injury of the DSB and lead to alterations in the DNA sequence and ultimately in gene expression.

(B) hCas9 D10A nickase: The wild-type endonuclease activity of Cas9 may, however, result in genome rearrangements that can lead to deleterious effects, e.g., selection against cells expressing wild-type Cas9 was observed (Qi et al., 2013). Furthermore, the generation of insertion/deletions (indels) at the target region as a consequence of DSB repair may lead to unwanted side-effects (Ketteler, 2012). To avoid those effects, Cong et al. used a mutant Cas9 nickase that only generates a nick in the genomic DNA at the target region, which in turn is repaired through high-fidelity homology-directed repair, rather than the error-prone Cas9 endonuclease-mediated non-homologous end-joining repair (Cong et al., 2013). The experiments to date suggest that this mutant Cas9 nickase shows similar targeting efficiencies compared to wild-type Cas9. Another advantage of the nickase system is that it can be used for generating knockout as well as knockin genotypes. This can be achieved by co-transfection of a recombination cassette with 5′- and 3′-flanking homology regions. However, knockout/knockin constructs based on the Cas9 nickase system require engineering of homologous recombination cassettes for each gene locus, thus making it technically challenging to implement on a large-scale.

(C) Catalytically dead dCas9: Qi et al. (2013) took a step further to generate a catalytically dead Cas9 lacking endonuclease activity. This resulted in gene silencing rather than gene editing of the target locus. This offers a significant advantage over classic RNAi-based silencing since mRNA synthesis is altered at an early stage of transcription by blocking RNA polymerase and transcript elongation whereas in RNA interference, the expressed synthesized mRNA is degraded. With this novel system, termed CRISPRi, the promoter region can also be targeted to efficiently knock down expression of the transcript. Moreover, Qi et al. (2013) showed that CRISPRi can be used to simultaneously repress multiple target genes (similar to chimeric hairpin constructs in RNAi, Gil-Humanes et al., 2010), which opens new strategies for large-scale profiling of genetic interactions in mammalian cells. The CRISPRi has enormous potential for gene silencing applications. Recently, it was shown that catalytic dead Cas9 can be fused to a general repressor or activator protein such as KRAB or VP16, respectively, and thereby result in highly efficient gene silencing or activation (Gilbert et al., 2013). Furthermore, an inducible silencing system has been described, thus expanding the versatility of this approach. It is conceivable that catalytic dead Cas9 and the associated sgRNA serves as genomic targeting tools, allowing genomic site-specific modifications. The versatility of this system is very attractive, allowing modifications that reach beyond the scope of RNAi libraries. Further studies are required to address whether silencing efficiency is similar to RNAi-mediated silencing.

Overall, the three described Cas9 systems show advantages depending on the application. For instance, wild-type hCas9 seems best suited to generate gene knockouts, nickase hCas9 D10A for gene replacement strategies and catalytically dead dCas9 for gene silencing.

## HIGH-THROUGHPUT SCREENING USING CRISPR/Cas9

Recent improvements have enabled the use of the CRISPR/Cas9 system as a novel tool to manipulate specific genomic regions. These developments have prompted the proposal to use this technology to set-up high-throughput screening approaches analogous to siRNA-mediated large-scale screening experiments (Qi et al., 2013). Next, we would like to consider some aspects that need to be addressed to enable the generation of large-scale CRISPR (crRNA) libraries.

(1) Design

(2) Coverage

(3) Efficiency of knockdown

(4) Off-target effects

(5) Delivery

### DESIGN

**FIGURE 1:**
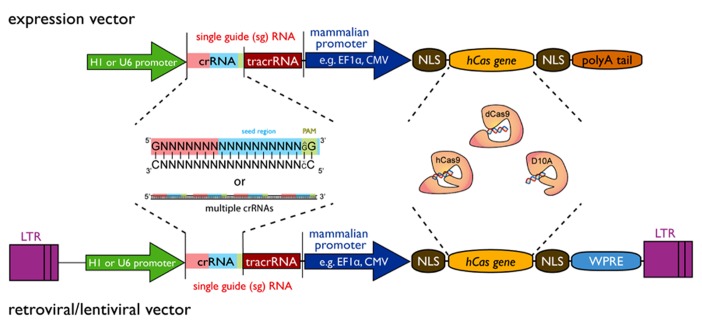
**Design principle of a CRISPR/Cas9 expression vector for construction of large-scale libraries.** The vector requires two minimal components, i.e., the single-guide RNA (sgRNA) sequence and the Cas9 gene that can both be expressed from a single-vector system. The sgRNA is composed of a variable crRNA and a constant tracrRNA. The gene sequence can be inserted by a simple adaptor cloning step in the 20 bp crRNA region. Furthermore, multiple target sequences of the same gene can be inserted to increase efficiency and reduce off-target effects. At the 3′-end of the crRNA sequence, a 2 bp (base pair) protospacer adjacent motif (PAM – green box) of the sequence GG is essential, but AG may be used to a lesser extend as well. A 12 bp seed region (blue box) at the 3′-end is required for sequence tar-geting with additional 8 bp at the 5′-end contributing to specificity (red box). sgRNA expression can be driven by a U6 promoter or alternatively by a H1 promoter. The Cas9 component is currently available in three different forms: as a wild-type (hCas9) or a mutant (D10A) version for gene editing purposes and a catalytically dead (dCas9) version for gene silencing approaches. Exp-ression of Cas9 can be driven by any mammalian expression promoter (e.g., EF1A, CMV, etc.) or retroviral promoter (LTR). For nuclear targeting, the Cas9 gene requires multiple nuclear localization signals (NLS) and general expre-ssion of Cas9 can be enhanced by inclusion of a woodchuck post-transcriptional regulatory element (WPRE) at the 3′-end. A polyA-tail is required for expression vectors, while it should be deleted from lenti-/retroviral expression vectors that have a polyA recognition sequence in their 3′-LTR. Variations of this design are possible with respect to the Cas gene used or further modifications such as tagging the Cas gene with GFP or NLSs in order to optimize Cas9 nuclear targeting.

Several possible configurations of the CRISPR/Cas9 system exist (see **Figure 1**). The most striking advantage for all of them is the simplicity by which a gene can be targeted. Two main components are required: first, a codon-optimized version of Cas9 endonuclease and second, the RNA components crRNA and tracrRNA. The DNA sequence encoding the crRNA has a length of about 20 nucleotides and is also known as protospacer. At the 3′ end the crRNA sequence must finish with a 2 bp (base pair) protospacer adjacent motif (PAM) of the sequence GG or AG. The crRNA sequence can be designed as complementary to the + or - strand of the target DNA region. As mentioned before it is possible to combine the crRNA sequence with the tracrRNA sequence in a sgRNA. Also CRISPR vectors for the expression of sgRNA and Cas9 from a single plasmid can be obtained from various sources via Addgene^1^. The generation of a genomic library for genome editing (using wild-type Cas9 or Cas9 nickase) or gene silencing (using catalytic inactive Cas9) can be achieved by a simple cloning of short DNA oligonucleotides into the respective CRISPR vectors. Protocols for plasmid construction and sub-cloning are freely available^2^^,^^3^. Protocols for automation of such cloning on a large-scale have previously been developed and applied to the generation of genome-wide shRNA libraries (Moffat et al., 2006). Briefly, synthesized oligonucleotide pairs coding for the desired crRNA can be annealed separately and ligated into the CRISPR vector. The ligations can be transformed into competent bacteria in a 96-well plate format. The resulting transformations from one plate can be pooled and plated onto an agar plate for robotic colony picking and plasmid sequencing. This process showed a remarkable efficiency when applied to the generation of genome-wide shRNA libraries (Moffat et al., 2006).

It should be noted that other Cas family members and Cas proteins from other organisms than *Streptococcus pyogenes *may also be suitable for gene editing purposes. This can have significant advantages in the selection of the target sequence as some Cas endonucleases have different requirements with regards to the PAM motif, which currently restricts the target efficiency of Cas9 to GG-PAM. For a review on sequence requirements of Cas proteins, please see Biswas et al., 2013.^4^ Furthermore, improvement of Cas9 and sgRNA expression and assembly, sgRNA-5′ and 3′ modifications or nuclear targeting will readily allow for improved CRISPR efficiency.

### COVERAGE

The main restriction for the design of sgRNA sequences is the PAM motif. There is strong selection for a GG motif, while the AG motif might be used to a lesser extent as well (Jiang et al., 2013). Accordingly, it was estimated that 1/8 of the genome hosts such a motif, thus enabling very high coverage of genetic regions. The Church lab has compiled a list of sites within the *Saccharomyces cerevisiae* and *H. sapiens* genome with regions suitable for incorporation into the sgRNA (Dicarlo et al., 2013; Mali et al., 2013). It was estimated that 40.5% exons of genes in the human genome have a perfect match to the *Streptococcus pyogenes* Cas9 PAM motif and ~190,000 sgRNA-targetable sequences were identified in total (Mali et al., 2013). Using other Cas9 family members that target regions with different PAM motifs will enable a good coverage of the whole-genome. It still needs to be established how to select the best suitable sequence for each gene.

### EFFICIENCY

The efficiency of gene silencing by various CRISPR/Cas9 systems has been observed to be variable and dependent on the cell type, as well as the guide RNA sequence. For instance, length and sequence complementarity of the crRNA as well as the position to which the crRNA binds within the gene locus, have been reported to affect silencing efficiency (Gilbert et al., 2013; Qi et al., 2013). Repression was inversely correlated with target distance to the transcription start site. Mali et al. observed a targeting efficiency of 10–25% in 293T cells, 13–38% in K562 cells, and 2–4% in induced pluripotent stem cells using wild-type Cas9 (Mali et al., 2013). Hwang et al. (2013) achieved a targeting efficiency of >80% in zebrafish. The CRISPRi system using catalytically inactive Cas9 achieved efficiencies of gene silencing of 46–63% in HEK293 cells and much higher in *E. coli *(Qi et al., 2013).

Overall, the efficiency of knockdown needs further improvement to about 70–80% in order to produce robust phenotypes. With improvements in transfection or lenti-/retroviral infection and expression protocols as well as sgRNA design principles, the outlook is quite promising that this will be possible. Further, the incorporation of a combination of multiple crRNA sequences into each construct might be a possibility to enhance the efficiency of knockdown.

### OFF-TARGET EFFECTS

The effect of off-target silencing has been evaluated in only a few examples. The minimal length of the base-pairing region within the crRNA is 12 bp (“seed region”), which can in some cases lead to significant binding to other regions of the genome. A recent study suggests that CRISPR-based genome editing has more off-target effects than other genome editing tools such as TALENs or ZFNs (Fu et al., 2013). In another study, 3-bp mismatches did not result in any detectable off-target effect for genome editing of the CCR5 locus (Cho et al., 2013). A more systematic study showed that single base and to a lesser extend two base mismatches are tolerated by SpCas9, thus potentially contributing to off-target effects (Hsu et al., 2013). The group presented algorithms to predict such off-target effects and therefore will enable the selection of target sequences with minimal off-target effects^5^. Another study that addressed the off-target effects of CRISPRi-mediated silencing using RNA-seq has shown that silencing is highly specific with minimal off-target effects (Gilbert et al., 2013). Further, combining two crRNAs drastically enhances silencing efficiency (Qi et al., 2013) and may reduce off-target effects significantly. It is conceivable that the use of different systems (wt vs. nickase vs. CRISPRi) and model organisms may result in different off-target behavior. Some studies to date suggest that CRISPR-mediated genome editing shows more off-target effects than other genome editing tools, but has similar or better target gene specificity than RNAi-based silencing. Clearly, more work needs to be done to evaluate the extent of off-target effects carefully.

### DELIVERY

The combination of both Cas9 endonuclease and the sgRNA in one plasmid construct enables the use of single plasmid transfection into most standard cell lines. For primary cells, specialized protocols for delivery such as those based on electroporation or microinjection may have to be used (Marine et al., 2012; Hwang et al., 2013). Transfection-based methods can be easily automated, which enables the use of the CRISPR/Cas9 system for high-throughput screening purposes. An alternative is the use of retro- or lenti-viral transduction, although one should keep in mind that there are biosafety considerations and extra care must be taken when handling humanized Cas9 endonuclease expression constructs. The use of short-term selection such as puromycin treatment can be included to enhance selection for stronger phenotypes (Wang et al., 2013). Longer selection and differentiation protocols can even enable the generation of whole organism-based silencing effects, for instance in zebrafish or mice (Hwang et al., 2013; Wang et al., 2013).

## CONCLUSION

In conclusion, the design of a whole-genome targeted sgRNA library is feasible provided that CRISPR/Cas components are further optimized to ensure high-confidence genome engineering. Clearly, more data will be generated in the coming months to evaluate carefully off-target effects and sequence requirements for efficient gene knockdown. Also, the assessment of other Cas9 proteins for genome editing and silencing will be an important step forward. It should be noted that gene knockout of essential genes may result in lethality effects, thus making the analysis of more subtle phenotypes difficult. In those cases, silencing strategies such as RNAi-mediated knockdown or CRISPRi-mediated gene repression may be beneficial. Overall, given the simplicity of use developments need to be encouraged for the design of CRISPR/Cas-based large-scale whole-genome loss-of-function screening applications. Current RNAi libraries are limited to three model organisms, i.e., human, rat, and mouse. Since CRISPR gene silencing works very well in multiple model organisms, the development of libraries for other model organisms should be particularly encouraged.

## Conflict of Interest Statement

The authors declare that the research was conducted in the absence of any commercial or financial relationships that could be construed as a potential conflict of interest.

## Footnotes

1

2

3

4

5 See prediction tool at
